# Molecular examination for *Coxiella burnetii* and *Brucella* spp. infections in Iranian women experiencing spontaneous miscarriage

**DOI:** 10.1186/s12879-024-09041-5

**Published:** 2024-02-07

**Authors:** Neda Baseri, Amir Hossein Omidi, Mina Latifian, Ehsan Mostafavi, Shahram Khademvatan, Navid Omidifar, Seyyed javad Seyyed Tabaei, Rasool Jafari, Shiva Zeinali, Ahmad Ghasemi, Saber Esmaeili

**Affiliations:** 1 Department of Epidemiology and Biostatics, Research Centre for Emerging and Reemerging Infectious Diseases, Pasteur Institute of Iran, Tehran, Iran; 2 National Reference Laboratory for Plague, Tularemia and Q Fever, Research Centre for Emerging and Reemerging Infectious Diseases, Pasteur Institute of Iran, Akanlu, Hamadan, KabudarAhang Iran; 3 Department of Bacteriology, Faculty of Medical Sciences, Tarbiat Modares University, Tehran, Iran; 4 Department of Medical Parasitology and Mycology, Urmia University of Medical Sciences, Urmia, Iran; 5 Cellular and Molecular Research Center, Cellular and Molecular Medicine Research Institute , Urmia University of Medical Sciences , Urmia , Iran; 6 Department of Pathology , School of Medicine, Shiraz University of Medical Sciences , Shiraz , Iran; 7 Department of Parasitology and Mycology, School of Medicine, Shahid Beheshti University of Medical Sciences, Tehran, Iran; 8 Department of Microbiology, Research Center of Reference Health Laboratories, Ministry of Health and Medical Education, Tehran, Iran

**Keywords:** *Coxiella burnetii*, *Brucella*, Spontaneous abortion, Miscarriage, Iran

## Abstract

**Background:**

Spontaneous miscarriage, a leading health concern globally, often occurs due to various factors, including infections. Among these, *Coxiella burnetii* and *Brucella* spp. may have adverse effects on pregnancy outcomes. While previous research has established a link between infections and spontaneous miscarriage, our study aimed specifically to investigate the presence of these two pathogens in abortion samples from women who experienced spontaneous miscarriages in Iran. Our study can add to the existing knowledge by focusing on Iran, a region with a high prevalence of *C. burnetii* and *Brucella* spp. As a result, it could provide a better understanding and unique insights into the relationship of these pathogens with spontaneous miscarriages in endemic regions.

**Methods:**

From March 2021 to March 2022, a total of 728 abortion samples (including placenta and cotyledon) were collected from 409 women who had experienced spontaneous miscarriages in the provinces of Tehran, Fars, and West Azerbaijan in Iran. The specimens included 467 Formalin-Fixed Paraffin-Embedded (FFPE) and 261 fresh frozen samples. After DNA extraction from abortion samples, the quantitative real-time PCR (qPCR) assay targeted a specific fragment of the IS*1111* and IS*711* elements for molecular identification of *C. burnetii* and *Brucella* spp., respectively. Furthermore, the qPCR assay employing specific primers for different species was used to determine the species of *Brucella*.

**Results:**

Among the studied women, 1 out of 409 (0.24%) samples tested positive for *Brucella* spp., specifically *Brucella melitensis*. There were no positive specimens for *C. burnetii*.

**Conclusions:**

Our study contributes to understanding the potential involvement of *Brucella* species in spontaneous infectious abortion within endemic regions. The identification of *B. melitensis* in this study highlights the need for further research in this area. However, while our results suggest a relatively low or zero identification of these pathogens in our sample population, this does not rule out the possibility of undetected infections. Therefore, it is critical to acknowledge the limitations of the molecular techniques used (qPCR), which may have potential limitations such as sensitivity and specificity. Moreover, because 64.15% of our samples were FFPE, the sensitivity of the qPCR test may be reduced. These raise concerns about the accuracy of the reported prevalence rates and the potential for false positives or negatives.

## Background

Miscarriage is defined as the spontaneous termination of pregnancy and is estimated to occur in approximately 15–20% of pregnancies. While the exact causes of miscarriage remain unclear, several common factors contribute to this occurrence, including genetic abnormalities, uterine abnormalities, maternal age, maternal overweight, hypertension during pregnancy, hormonal imbalances, immunological factors, history of miscarriage, smoking, alcohol consumption, psychological stress, and infections [1–3]. 15% of early abortions and 66% of late abortions are caused by infections [1]. Among the infections, some pathogens have been implicated in increasing the risk of spontaneous miscarriages. For instance, infections caused by bacteria such as *Listeria monocytogenes*, *Ureaplasma urealyticum*, and *Chlamydia trachomatis* have been associated with adverse pregnancy outcomes, including miscarriages [4]. Additionally, certain viral infections like cytomegalovirus (CMV) and herpes simplex virus (HSV) have also been linked to an increased risk of miscarriages [5]. These infections can lead to inflammation, placental damage, and ultimately fetal loss [6, 7]. However, the mechanisms responsible for infectious miscarriages are diverse and can involve direct fetal infection, placental injury, and severe maternal illness. In many cases, despite the presence of elevated levels of maternal inflammatory markers or histological evidence of chorioamnionitis, no specific pathogenic agents have been identified. Intracellular bacteria, such as *Coxiella burnetii* and *Brucella*, which cannot be easily detected using conventional culture media routinely used for pathogen detection in clinical specimens, may play a significant role as etiological agents of miscarriages [1, 4]. However, their contribution is likely underestimated [1]. Advancements in modern diagnostic techniques, particularly molecular methods, have led to increased identification of infectious agents that can cause pregnancy complications, including spontaneous miscarriages [4].

*Coxiella burnetii* is a Gram-negative bacterium and the etiological agent of the zoonotic disease known as Q fever. The main way of contracting Q fever is through the inhalation of aerosols and particles contaminated with *C. burnetii*. Direct contact and the consumption of unpasteurized dairy products are less common methods of transmitting the infection to humans [8, 9]. Q fever can cause various clinical manifestations in humans, ranging from asymptomatic to acute Q fever (resembling flu-like symptoms, pneumonia, and hepatitis) and chronic Q fever (endocarditis) [10]. During pregnancy, both acute and chronic Q fever can lead to complications such as premature delivery, growth retardation, fetal abnormalities, low birth weight, stillbirth, and abortion [11]. *C. burnetii* not only colonizes but also multiplies in various parts of the body, including the uterus, placenta, monocytes, macrophages, and human trophoblastic cells [12]. The prevalence of *C. burnetii* among pregnant women in Iran has been reported to be approximately 30% [13]. Q fever is endemic in Iran, and acute Q fever and Q fever endocarditis cases have been reported in various studies [14, 15]. The incidence of Q fever during pregnancy is likely underestimated, and there is limited information available regarding the role and prevalence of this bacterium in pregnancy complications such as miscarriages. Hence, it is of utmost importance to investigate the presence of *C. burnetii* in miscarriages, especially considering the high seroprevalence among livestock and milk in Iran [16, 17].

*Brucella* is a Gram-negative bacterium that causes the disease known as brucellosis. Humans typically contract the disease through direct contact with infected animals, consuming contaminated animal products, or inhaling airborne agents [18]. There are various *Brucella species*, such as *B. abortus*, *B. melitensis*, *B. suis*, and *B. canis* [19], all of which can cause brucellosis in humans. This disease can lead to complications during pregnancy, including miscarriage, premature birth, contagious or neonatal brucellosis, intrauterine infection, or intrauterine fetal death [20]. Brucellosis is an endemic disease in Iran and has been reported in different regions of the country; the prevalence rate varies from 98 to 130 per 100,000 population [21, 22]. In endemic areas, the prevalence rates of brucellosis in pregnant women range from 1.3 to 12.2% [23]. A study in Iran found that 53% of pregnant women with brucellosis experienced spontaneous miscarriages [24]. In Iran, there is a lack of research on the genetic proof of *Brucella* in women who have had abortions, making it imperative to examine the presence of *Brucella* in these women.

While the role of *C. burnetii* and *Brucella* spp. in spontaneous miscarriages is well-documented, our study focuses specifically on Iran, a region with a high prevalence of these pathogens, providing unique insights into their impact on spontaneous miscarriages. Therefore, this study aimed to investigate the presence of *Brucella* and *C. burnetii* in abortion samples from women with a history of abortion in Iran, as these infectious and miscarriage abortions are prevalent health issues in the country due to the endemic nature of both *Brucella* and *C. burnetii*.

## Methods

### Sample collection

**Fig. 1:**
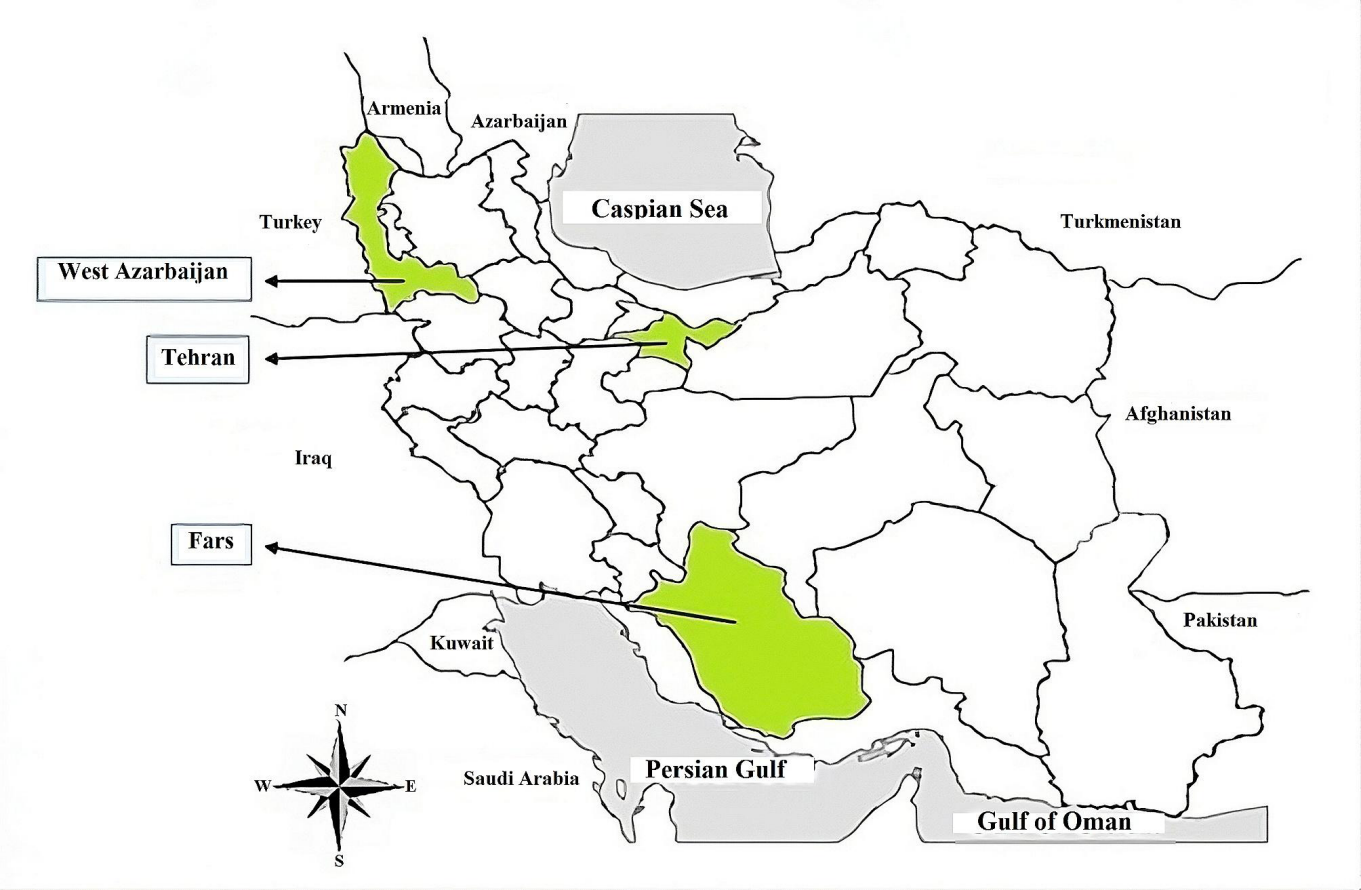
Q1The provinces in this study: Tehran, Fars, and West Azerbaijan

We conducted a completely random sampling method between March 2021 and March 2022 and collected 728 abortion tissue samples, including placenta and cotyledon, from 409 pregnant women in Tehran, West Azerbaijan, and Fars provinces (Fig. 1). The choice of these provinces was based on their geographic representation and accessibility of samples from biobanks and rural areas.

Our sample collection strategy was designed to maximize the diversity of our study population. Therefore, we collected samples from both urban and rural settings to account for differences in exposure to the pathogens of interest.

In this study, 261 fresh frozen samples were obtained from the biobanks of Urmia University of Medical Sciences (West Azerbaijan Province) and Shahid Beheshti University of Medical Sciences (Tehran Province), as well as from rural women with a history of abortion. We also included 467 formalin-fixed and paraffin-embedded (FFPE) blocks of placenta samples from rural women, which were accessible at Shiraz University of Medical Sciences in Fars Province.

Participants were selected based on having experienced a spontaneous miscarriage. They were required to provide informed consent before participation. The age of the participants ranged from 15 to 43 years. Age was chosen as a criterion because older ages have been associated with a higher risk of spontaneous miscarriages.

### Sample preparation

The fresh frozen samples were prepared by cutting up to 25 mg into thin sections using a sterile razor blade.

For FFPE samples, the sections were first deparaffinized using xylene. Deparaffinization removes the paraffin wax from the tissue sections, which is necessary for subsequent DNA extraction steps. After deparaffinization, the samples were treated with ethanol solutions of varying concentrations (100, 90, 80, and 60%, respectively) for xylene elimination and tissue hydration. This step helps to remove the residual xylene from the tissue samples and to hydrate the tissue, making it easier to extract DNA. Finally, up to 25 mg of these tissues were stored for DNA extraction.

### DNA extraction

DNA was extracted from the prepared tissue samples using the Animal Tissue DNA Isolation Kit (Viragen Co., Iran) according to the manufacturer’s instructions. Finally, the absorbance at 260 nm, 280 nm, and 230 nm of the extracted DNA was evaluated for quantity and quality using a NanoDrop (Epoch BioTek, USA). Absorbance ratios of 260/280 nm ~ 1.8 and 260/230 nm ~ 2.0-2.2 were considered good quality.

### **Identification of*C. burnetii*and*Brucella*spp.**

To identify *C. burnetii* and *Brucella* spp., TaqMan quantitative real-time PCR (qPCR) was conducted on extracted DNA samples using the primers and probes that were previously published and designed [25, 26] to target highly conserved regions of the IS*1111* and IS*711* genes, respectively. The qPCR was carried out in a final volume of 20 µl, containing 10 µl of commercial master mix 2x Real Q Plus Master Mix for Probe (Ampliqon Co., Denmark), 900 nmol of forward primer, 900 nmol of reverse primer, 200 nmol of probe (Table 1), 4 µl (1–100 ng) of DNA template, and distilled water to reach the final volume. The amplification process was conducted in a Corbett 6000 Rotor-Gene system thermocycler (Corbett, Victoria, Australia) with the following program: initial denaturation at 95 °C for 10 min, followed by 45 cycles at 95 °C for 15 s and 60 °C for 60 s.

To ensure the reliability of our results, several validation steps were undertaken. Firstly, all qPCR assays were evaluated for their analytical sensitivity using serial dilutions of known positive DNA samples, and the lower limit of detection (LOD) was determined. Secondly, positive and negative controls were included in each qPCR run. Distilled water (no template DNA) was used as a negative control, while the *C. burnetii* Nine Mile RSA493 strain and a known positive DNA extracted from clinical strain for *B. abortus* served as positive controls for *C. burnetii* and *Brucella* spp., respectively. A sample was considered positive for a particular pathogen if it showed amplification with a quantification cycle (Cq) value below the LOD and if the amplification curve matched the expected pattern. Samples yielding Cq values above the LOD were considered negative or inconclusive.

### *Brucella* species identification

After performing the qPCR analysis to identify the *Brucella* genus in the previous step, a second qPCR assay was conducted using specific primers and probes to detect the different *Brucella* species, such as *B. melitensis* and *B. abortus* (Table 1). The PCR conditions remained consistent with those mentioned in the previous section.

**Table 1:** Primer and specific probes for the detection of *Coxiella burnetii* and *Brucella* spp., *Brucella abortus*, and *Brucella melitensis*

Species	Target	Primer	Sequencing primers (5’→3’)	Size fragments (bp)	Reference
***C. burnetii***	IS*1111*	Forward	5’-AAAACGGATAAAAAGAGTCTGTGGTT-3’	70	[25]
		Reverse	5’-CCACACAAGCGCGATTCAT-3’		
		Probe	5’-6-FAM-AAAGCACTCATTGAGCGCCGCG-TAMRA-3’		
***Brucella*spp**.	IS*711*	Forward	5’-GCTTGAAGCTTGCGGACAGT-3’	63	[26]
		Reverse	5’-GGCCTACCGCTGCGAAT-3’		
		Probe	5’-FAM-AAGCCAACACCCGGCCATTATGGT-TAMRA-3’		
***B. melitensis***	*BMEII0466*	Forward	TCGCATCGGCAGTTTCAA	67	[27]
		Reverse	CCAGCTTTTGGCCTTTTCC		
		Probe	TEX-CCTCGGCATGGCCCGCAA-BHQ-2		
***B. abortus***	*BruAb2_0168*	Forward	GCACACTCACCTTCCACAACAA	81	[27]
		Reverse	CCCCGTTCTGCACCAGACT		
		Probe	HEX-TGGAACGACCTTTGCAGGCGAGATC-BHQ-1		

## Results

Out of the 728 abortion samples, there were 50 frozen samples from Tehran Province, 211 samples from West Azerbaijan, and 476 FFPE samples from Fars Province belonging to 148 aborted women. The average (± SD) age of the women with spontaneous abortion was 29 years (range: 15–43 years).

In fresh frozen samples, 111(51.39%) were collected from urban areas, while 105 (48.61%) were from rural regions. However, all 467 FFPE samples were originated from rural locations.

Regarding the stage of pregnancy, we currently have information available only for patients with fresh frozen samples, and not for FFPE samples. In our study, the majority of spontaneous abortion cases in patients with fresh frozen samples occurred in the first trimester of pregnancy (week 1 - week 12), making up 53.95% of cases. In the second trimester (week 13 - week 28), there were 116 cases (53.95%). Interestingly, there were no cases of spontaneous abortion recorded in the third trimester (week 29 - week 40).

Among the 728 samples obtained from 409 women, only one sample was positive for *Brucella* spp. using the qPCR method. This sample had a Cq value of 28.5. In a second specific qPCR assay, the positive sample was identified as *B. melitensis* with a Cq value of 23.36. The sample belonged to a 23-year-old worker woman with a weight of 54 kg who experienced a spontaneous abortion on day 83 (first trimester) of her pregnancy. This woman resided in a rural area of West Azerbaijan Province and had no history of previous miscarriages or underlying diseases.

Furthermore, the present study revealed that none of the samples tested positive for *C. burnetii*.

## Discussion

In this study, the prevalence of *C. burnetii* and *Brucella* was investigated in samples from miscarriages. Miscarriage is a common complication of pregnancy that can occur frequently or sporadically [28]. Some intracellular bacteria, including *L. monocytogenes*, *C. trachomatis*, *C. burnetii*, and *Brucella*, have been found to affect embryonic units and cause serious diseases in both the mother and fetus [4]. For instance, a study conducted in Switzerland from 2006 to 2009 revealed that the prevalence of IgG against *C. trachomatis* was more common in the group experiencing miscarriages (15.2%) compared to the control group (7.3%; *p* = 0.018). Additionally, the DNA of *C. trachomatis* was more frequently detected from the products of conception or placenta in women who had miscarriages (4%) compared to those in the control group (0.7%; *p* = 0.026) [29]. However, few studies have been conducted on Q fever infections in pregnant women and the associated abnormal and complex complications. Adverse pregnancy outcomes resulting from Q fever infections in pregnant women are typically caused by vascular thrombosis, which leads to placental insufficiency and abortion. Studies have also reported direct infection of the fetus [30]. Serological studies of *C. burnetii* among pregnant women living in rural areas with direct contact with livestock were found to be 29.3% in southwestern and northern Iran and 48.4% in western Iran [13, 31]. However, our study did not detect any *C. burnetii* infections using the molecular method.

Acute Q fever infection during the first trimester of pregnancy significantly increases the risk of both the mother and fetus developing chronic Q fever infection. Furthermore, an asymptomatic infection during pregnancy can progress to a chronic state, increasing the chances of reinfection in future pregnancies [32, 33].. In Denmark, a study found a significantly higher seroprevalence rate (47%) of Q fever among pregnant women who were exposed to livestock than among nonexposed women (4.8%) [34].

Several studies have investigated the prevalence of *C. burnetii* in abortion samples from pregnant women using molecular methods. One study conducted in Turkey on 51 placental samples found no detection of *C. burnetii* [35]. Similarly, a study in France on 246 placenta samples also reported no infection with this pathogen [36], which is consistent with our findings. However, a study in Algeria reported a positive rate of 0.55% in placental samples [37]. In Iraq, a molecular study reported that 17.02% of blood samples from aborted women were positive for *C. burnetii* [38]. Many studies have focused on the prevalence of Q fever in pregnant women using serological methods. In two separate studies conducted in Turkey, the seroprevalence of Q fever among pregnant women was found to be 20.7% and 14%, respectively [35, 39]. In Iran, the seroprevalence of *C. burnetii* infection in pregnant women was found to be 22% in the southwest (Ahvaz City) and 36.5% in the north (Parsabad City). Furthermore, the overall prevalence of *C. burnetii* infection in women with a history of abnormal pregnancy (39.8%) was significantly higher than that in women with normal pregnancies (23.8%) [13]. Additionally, a study conducted among rural pregnant women in Khorramabad (western Iran), who were in contact with livestock, reported a seroprevalence of 48.4% for *C. burnetii* [31]. However, a possible explanation for the non-detection of *C. burnetii* in abortion samples during this study could be due to the exclusion of specific infection foci in the examined tissue samples. In addition, it is plausible that the infection was latent or had already resolved by the time of examination, rendering the molecular test insufficient for diagnosis.

Although the presence of erythritol in the placenta of livestock acts as a growth factor for *Brucella*, cases of abortion have also been reported in humans [40–42]. The first case of brucellosis in a pregnant woman was reported in 1908 in a rural area [43]. In Egypt, the seroprevalence of this disease in pregnant women in endemic areas was found to be 12% [44].. In our study, a positive case of *Brucella* was only reported in the abortion sample of a pregnant woman living in a rural area. This positive sample was classified as *B. melitensis*, which causes brucellosis in humans and is known to primarily cause abortion and stillbirth [19]. In Saudi Arabia, a study reported a 3.5% prevalence of brucellosis in women living in rural areas. Furthermore, the incidence of abortion in pregnant women with *Brucella* titers greater than 1:160 was found to be 17.6%, while the incidence of abortion in women with a lower titer was significantly lower at 7.7% [45]. In 1954 in Spain, among 200 pregnant women infected with *B. melitensis*, 10% of these women experienced abortion in the first trimester (34). In Iran, the first study on human spontaneous abortion conducted in 1974 included 51 women from endemic regions in Isfahan Province who had abortions during their second trimester of pregnancy. Among these patients, six showed clinical and laboratory evidence of brucellosis infection, with five positive cultures of fetal remains and placenta samples [46].

In a study in Turkey, 24.1% of infected pregnant women suffered a miscarriage, whereas only 7.6% of the control group tested positive for brucellosis [47]. In Pakistan in 2016, the seroprevalence of *Brucella* in pregnant women was found to be 5.8%, with a significant rate of positive cases in rural areas. In addition, seroprevalence was reported to be 14.6% in women who experienced their first abortion, 12.5% in women in contact with infected animals, and 15.8% in women with a history of multiple abortions [20]. In a study in London in 2009, the seroprevalence of *C. burnetii* and *Brucella* among 438 pregnant women was found to be 4.6% and 0.5%, respectively [48]. Finally, in another study in Pakistan in 2021, it was discovered that 25.5% of women with a history of abortion were positive for *Brucella* using molecular methods. The predominant species identified in this study was *B. abortus* [49].

Since spontaneous miscarriages can be influenced by a multitude of factors beyond infections, such as *B. melitensis*, it should be noted that in this study and most studies, no adjustment was made for several risk factors that could be considered potential confounders. For instance, hypertensive disorders of pregnancy (HDP), which affect around 5–10% of pregnancies worldwide, can lead to complications such as fetal loss [50]. A study conducted in the Southern United States from 2000 to 2012 found that women with at least one fibroid were more likely to experience a miscarriage compared to those without fibroids. The odds ratio was 4.33 for women aged 35 years and above [51]. In addition, one study found that Polycystic ovary syndrome (PCOS), a common endocrine disorder, was present in 59.8% of women with recurrent miscarriages [52]. Our study was also limited by restricted access to patients’ medical records, a crucial limitation that impacted the comprehensive gathering and analysis of data. Consequently, multiple potential risk factors were not considered during the adjustment process.Therefore, it should be noted that other potential causes of spontaneous miscarriages, make it challenging to contextualize the significance of infection among other known risk factors.

## Conclusion

In this study, we were unable to detect any positive samples for *C. burnetii*, although only one sample was found to be infected with *B. melitensis*. However, our results should not be interpreted as ruling out the possibility of miscarriage in pregnant women due to infection with these two pathogens. Acknowledging the limitations of qPCR for identification, it is important to note that the technique may have potential limitations in terms of sensitivity and specificity. False positives or negatives are possible, and further research would be valuable to validate our findings using alternative diagnostic methods. Moreover, because a significant proportion (64.15%) of our samples were derived from FFPE, it could contribute to a possible reduction in the sensitivity of the qPCR test used in this study. It is crucial to consider these factors when interpreting the results of the study. In addition, the study was conducted on a relatively small sample size. Therefore, to confirm our findings and increase statistical power, future studies should consider recruiting a larger sample size more quality and suitable samples, and additional laboratory tests. It is recommended that pregnant women residing in endemic areas for *Brucella* undergo screening and be monitored throughout their pregnancy using serological tests to ensure prompt identification and treatment. It is important to note that infection in pregnant women can be asymptomatic, which poses a challenge for timely diagnosis and prevention of adverse pregnancy outcomes. Consequently, healthcare systems in endemic areas should prioritize the management of these diseases.

## Data Availability

All data generated or analyzed during this study are included in this published article.

## Abbreviations

*C. burnetii*: *Coxiella burnetii*
qPCR: quantitative real-time PCR
*B. melitensis*: *Brucella melitensis*
*B. abortus*: *Brucella abortus*
FFPE: Formalin-fixed and paraffin-embedded
Cq: Quantification cycle
